# Automated VMAT planning for short-course radiotherapy in locally advanced rectal cancer

**DOI:** 10.1371/journal.pone.0325567

**Published:** 2025-06-09

**Authors:** Qiong Zhou, Liwen Qian, Chong Shen, Xinyan Bei, Gaojie Liu, Xiaonan Sun

**Affiliations:** Sir Run Run Shaw Hospital, School of Medicine, Zhejiang University, Hangzhou, ZheJiang, China.; Northwestern University Feinberg School of Medicine, UNITED STATES OF AMERICA

## Abstract

**Purpose:**

This study aims to develop a fully automated VMAT planning program for short-course radiotherapy (SCRT) in Locally Advanced Rectal Cancer (LARC) and assess its plan quality, feasibility, and efficiency.

**Materials and methods:**

Thirty LARC patients who underwent short-course VMAT treatment were retrospectively selected from our institution for this study. An auto-planning program for neoadjuvant short-course radiotherapy (SCRT) in LARC was developed using the RayStation scripting platform integrated with the Python environment. The patients were re-planned using this auto-planning program. Subsequently, the differences between the automatic plans (APs) and existing manual plans (MPs) were compared in terms of plan quality, monitor units (MU), plan complexity, and other dosimetric parameters. Plan quality assurance (QA) was performed using the ArcCHECK dosimetric verification system.

**Results:**

Compared to MPs, the APs achieved similar target coverage and conformity, while providing more rapid dose fall-off. Except for the V5Gy dose level, other dosimetric metrics (V25 Gy, V23 Gy, V15 Gy, Dmean, etc.) for the small bowel were significantly lower in the AP compared to the MP (p < 0.001). Additionally, the dosimetric parameters for the bladder, pelvic marrow, and femoral head were also lower in the AP, except for the V25Gy for the bladder. The MUs of the AP were approximately 4% lower than those of the MP. The AP showed high consistency in dosimetric parameters across five organs at risk (OARs).

**Conclusion:**

We developed a fully automated, feasible SCRT VMAT planning program for LARC. This program significantly enhanced plan quality and efficiency while substantially reducing the dose to OARs.

## Introduction

Colorectal cancer (CRC) is the second leading cause of cancer mortality worldwide, with an estimated 903,859 deaths in 2022, of which rectal cancer accounted for 38% of the CRC deaths [1]. Preoperative concurrent chemoradiotherapy in combination with total mesorectal excision is regarded as the standard treatment for locally advanced rectal cancer (LARC) [2]. Short-course radiotherapy (SCRT) and long-course radiotherapy (LCRT) are two common neoadjuvant radiotherapy regimens that are similarly effective in promoting tumor regression and reducing local recurrence rates [3–5]. However, SCRT has become increasingly favored in the treatment of LARC because it enhances patient compliance and shortens the treatment duration [6,7]. As SCRT constitutes a hypofractionated treatment approach, organs at risk (OARs) are more sensitive to the biological effects associated with dose variations. Therefore, in neoadjuvant radiotherapy planning for LARC, it is imperative to ensure adequate target volume coverage while rigorously limiting the radiation exposure to OARs, such as the bladder and small bowel, to mitigate the risk of radiation-induced toxicity.

Volumetric Modulated Arc Therapy (VMAT) is widely used in the clinical treatment of rectal cancer due to its high precision and optimal dose distribution [6,8,9].However, traditional VMAT treatment planning heavily relies on the experience of dosimetrists and an iterative trial-and-error process, which is time-consuming, labor-intensive, and fraught with uncertainty [10–13].To reduce workload and standardize the quality of treatment plans, various auto-planning approaches have been introduced in recent years, such as knowledge-based planning (KBP) and protocol-based automated iterative optimization (PB-AIO) [14].

KBP constructs dose prediction models (DVH) by leveraging high-quality historical planning data to guide the planning design for new patients. Its performance is highly dependent on the quantity, quality, and consistency of the training data [15,16]. In contrast, PB-AIO begins with a predefined template and progressively improves plan quality based on optimization feedback [17]. Given that its optimization approach closely resembles traditional manual planning, PB-AIO has been extensively studied in various fields, including head and neck [18–20], rectum [21,22], and chest [23]. However, research on auto-planning for SCRT in LARC remains limited. The unique pelvic anatomy and the specific dose and optimization requirements of LARC-SCRT necessitate site-specific validation to assess the clinical applicability of auto-planning.

The Direct Machine Parameter Optimization (DMPO) algorithm in the RayStation treatment planning system (TPS) significantly enhances both the efficiency and precision of radiation therapy planning. The DMPO algorithm directly optimizes physical parameters based on the current dose distribution, streamlining the optimization process and minimizing errors, resulting in more accurate treatment plans [24,25].

This study aims to develop a fully automated VMAT planning program for SCRT in LARC using the RayStation scripting platform integrated with the Python environment. While previous studies, such as Hirotaki et al. [26], have reported similar auto-planning approaches for conventionally fractionated VMAT in LARC using RayStation scripting, this study uniquely focuses on the application of automated VMAT planning specifically for SCRT, which involves distinct target volumes, clinical constraints, and planning criteria compared to conventional fractionation schemes. We compared the plan quality and delivery efficiency of these automated plans (APs) with manual plans (MPs) designed using the Monaco TPS (Elekta AB, Stockholm, Sweden) to evaluate the feasibility and practicality of implementing AP in clinical applications.

## Materials and methods

### Patient selection

Thirty patients (median age, 61 years; 77% male) with clinically staged T3a–T4a rectal adenocarcinoma who underwent neoadjuvant SCRT between January 2022 and November 2023 were retrospectively enrolled. All patients underwent treatment planning based on prone-position CT simulation with a 3-mm slice thickness. All VMAT plans used the same prescribed dose of 25 Gy in 5 fractions for PTV. Patients with a history of LCRT or those who developed metastatic disease during treatment were excluded. This study was approved by the Ethics Committee of Sir Run Run Shaw Hospital, Zhejiang University School of Medicine (Approval No. 2024-2147-01) and was conducted in accordance with the ethical standards of the Declaration of Helsinki. Patient consent was waived by the Ethics of Sir Run Run Shaw Hospital, Zhejiang University School of Medicine Committee due to the anonymity of the data. The data for this retrospective analysis were collected and analyzed between April and July 2024. The detailed experimental protocol is available on protocols.io and can be accessed at https://dx.doi.org/10.17504/protocols.io.q26g7357qvwz/v1.

### Contouring

The target volume and OARs were delineated by the same physician in accordance with established guidelines [27,28] and following multidisciplinary team (MDT) discussions [7].The delineated structures included the gross tumor volume (GTV), clinical target volume (CTV), the planning target volume (PTV) generated by expanding the CTV by 0.3 cm. The OARs included the small bowel, bladder, bilateral femoral heads, and pelvic marrow.

### Clinical goals

The following constraints were met for treatment planning [29]:

PTV: V25Gy ≥ 95%, Dmax<110%(27.5Gy)

Small bowel: V10Gy<200cc, V18Gy<110cc, V23Gy<85cc

Bladder: V25Gy<5%, V21Gy<15%

Femoral head: V12.5Gy<11%

pelvic marrow as low as reasonably achievable, avoiding hotspots.

### Manual planning

The MP was generated using the Monaco V6.00.11 TPS and Elekta Infinity linear accelerator (linac) by the same medical dosimetrist with over five years of experience. All plans were clinically approved and reviewed by senior radiation oncologists. The treatment plan utilized VMAT technology, with a dose rate of 1400 MU/min and 6 MV -flattening-filter-free (6 MV-FFF). The plan involved two arcs (204° ~ 156°, 156° ~ 204°), with a dose calculation grid size of 0.25 cm, and the overall plan uncertainty was 0.7%.

The dose calculation employed the XVMC algorithm of Monaco V6.00.11, based on the Voxel Monte Carlo (VMC) code, which enhances computational speed by utilizing a simplified multiple scattering distribution and energy cutoff. At the same time, it simulates radiation transport within the patient’s body to ensure both efficiency and accuracy [30,31].

### Auto-planning

The AP was based on RayStation 9A TPS (RaySearch Medical Laboratories AB, Stockholm, Sweden), with the planning program written in Python v2.7. The AP employed the same linac, beam angles, beam energy, calculation grid, and uncertainty parameters as the MP. Similar optimization objectives were applied based on the same set of dose constraints. The dose calculation for the AP used the Monte Carlo (MC) algorithm in RayStation 9A, which incorporated an EGSnrc-based MC photon algorithm and performed GPU-based direct calculations, significantly improving computational efficiency. The latest MC code in RayStation 9A delivers high-precision dose calculations in complex anatomical regions [32].

#### DMPO optimization algorithm.

The DMPO optimization algorithm consists of two main steps: flux optimization and the optimization of MLC at the field control points and other physical parameters. The key advantage of this algorithm is that it enables direct iterative optimization of physical parameters based on the current dose distribution, without needing to convert dose results into field control point parameters, thereby improving the accuracy of dose calculations.

The optimization was performed using RayStation’s built-in DMPO algorithm and standard optimization functions. The automation script developed for this study systematically selected and adjusted these functions and parameters in a structured, iterative manner (S1 File).

The specific optimization steps are as follows:

(1) Initial Setup: Create the VMAT plan, including auxiliary structures, beam, and planning goal, along with and optimization objectives (initial objectives are set with relatively relaxed). The initial template used for generating these APs is provided in S2 File.
(2) First Optimization: Perform 30 steps of flux optimization, adjusting objectives based on feedback values. Then continue with further optimization.
(3) Target and OAR Evaluation: Evaluate the feedback values for the target area and OARs. If the convergence conditions (threshold) are not met, tighten the objectives and continue the optimization process.
(4) Cycle Optimization: If the maximum number of cycles (3) has not been reached, continue optimization. Each cycle involves 100 iterations, normalized to the prescription dose.
(5) Optimization Completion: Optimization terminates when all feedback values from the objectives exceed the preset threshold (e.g., $4\times 10^{-4}$, and the number of cycles does not exceed 3. If the cycle count exceeds 3, reset the optimization results and restart the optimization process to prevent excessive iterations that could increase plan complexity.

**Fig 1:**
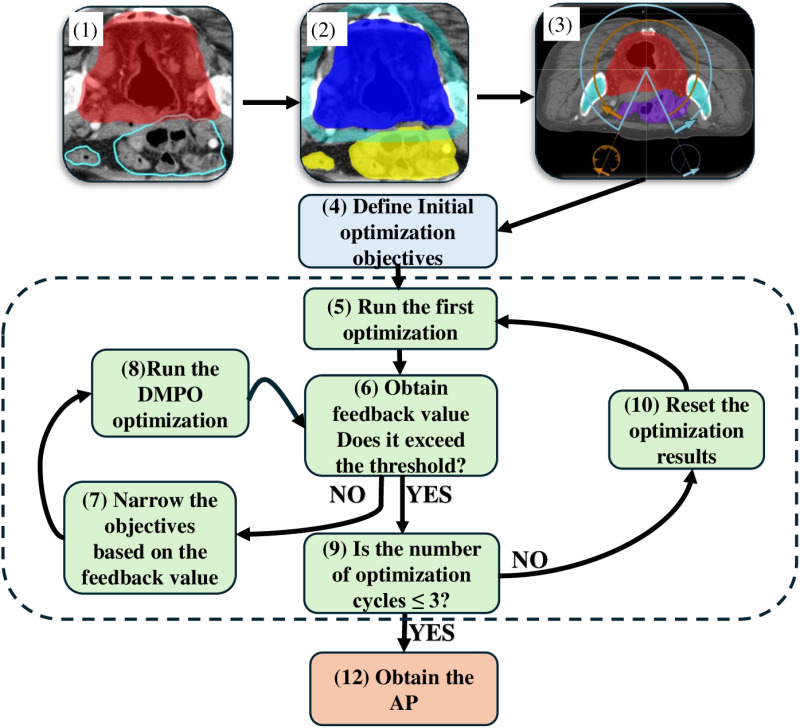
The workflow of the AP process.

Fig 1 presents the detailed workflow of the entire AP process.

All APs were simulated on the TPS but were not delivered in clinical practice. The complete dataset is available in S3 File.

The raw beam data— including percentage depth dose, off-axis ratio, output factor, and related parameters—were collected by vendor-certified physicists. The same dataset was used for beam modeling and commissioning in both TPSs. Acceptance testing was subsequently performed by experienced medical physicists at our institution in accordance with AAPM MPPG 5.a guidelines. This retrospective study utilized clinically delivered treatment plans that were originally generated using the Monaco TPS. The RayStation TPS was introduced at a later stage, and its auto- planning functionality was employed solely for research purposes. Although the two TPSs adopt different optimization algorithms, both use the MC dose calculation engine to ensure dosimetric consistency and clinical equivalence.

### Plan evaluation and statistical analysis

Plan quality was assessed based on dosimetric parameters for the PTV and OARs, including the maximum dose (D2%), minimum dose (D98%), mean dose (Dmean), Homogeneity Index (HI) [33], Conformity Index (CI) [33], Gradient Index (GI) [34], and monitor units (MU). In addition, the edge metric complexity (M) is used to quantify the complexity of each plan [35]. Theoretically, its value ranges from 0 to positive infinity, with typical clinical values between 0.05 and 0.30 mm⁻¹. Lower M values indicate simpler and larger field apertures, whereas higher M values reflect increased geometric complexity and irregular aperture shapes. Additionally, the absolute volumes or percentages of the small bowel, pelvic marrow, bladder, and femoral head receiving doses of 25 Gy, 20 Gy, 15 Gy, 10 Gy, and 5 Gy, as well as the mean dose (Dmean), were assessed. To assess work efficiency, the planning time from design optimization to completion for both AP and MP was recorded. Plan QA of all APs and MPs was performed by ArcCHECK® QA system (Sun Nuclear Corporation, Melbourne, FL, USA).

Normality of all data was assessed using the Shapiro-Wilk (S-W) test. For data that followed a normal distribution, group differences were analyzed using the paired t-test, with statistical significance defined as p < 0.05. For continuous variables that did not follow a normal distribution, the Wilcoxon signed-rank test was applied, with a significance threshold set at 0.05.

## Results

### Target dose coverage and dosimetric comparison

All dosimetric parameters for the PTV and OARs shown in Table 1 were evaluated. Both the AP and MP met the clinical dose constraints. The mean PTV volume for the 30 patients was (792.7 ± 141.94) cc. With 100% of the prescription dose covering 95% of the PTV, the AP showed higher D2%, Dmean, and HI compared to the MP, while D98%, CI, and GI were lower in the AP than those in the MP, with statistically significant differences (p < 0.001). The PTV homogeneity and conformality were similar between the AP and MP, and the AP demonstrated a more rapid dose fall off (2.96 VS. 3.45).

**Table 1:** Comparison of dosimetric parameters between AP(RayStation) and MP(Monaco).

Variable	Parameter	AP	MP	Test_Type	*P*
**PTV**	D_2%_(cGy)	2741.6 ± 16.88	2686.8 ± 24.33	Wilcoxon SRT	<0.001
	D_98%_(cGy)	2436.9 ± 9.41	2465.7 ± 11.07	Wilcoxon SRT	<0.001
	D_mean_(cGy)	2625.3 ± 13.38	2591.5 ± 14.43	Wilcoxon SRT	<0.001
**Small bowel**	D_mean_(cGy)	789.82 ± 111.03	855.68 ± 148.24	Paired t-Test	0.00167
	D_max_(cGy)	2471.3 ± 176.38	2554.9 ± 107.29	Wilcoxon SRT	<0.001
	V_5_ (cm^3^)	312.09 ± 127.03	306.73 ± 100.58	Paired t-Test	n.s.
	V_10_(cm^3^)	113.44 ± 47.13	133.03 ± 37.95	Paired t-Test	0.00871
	V_15_(cm^3^)	27.65 ± 18.69	39.76 ± 19.91	Wilcoxon SRT	<0.001
	V_18_(cm^3^)	0.44 ± 1.27	0.74 ± 1.24	Wilcoxon SRT	<0.001
	V_23_(cm^3^)	13.92 ± 12.23	21.63 ± 14.13	Wilcoxon SRT	<0.001
	V_25_(cm^3^)	3.18 ± 4.16	5.99 ± 5.23	Wilcoxon SRT	<0.001
**Bladde**	D_mean_(cGy)	889.46 ± 85	913.74 ± 132.04	Paired t-Test	n.s.
	V_5_(%)	77.68 ± 11.98	81.09 ± 17.61	Paired t-Test	0.03
	V_10_(%)	32.87 ± 6.29	33.75 ± 10.94	Paired t-Test	n.s.
	V_15_(%)	12.13 ± 4.78	13.34 ± 6.01	Paired t-Test	n.s.
	V_25_(%)	0.19 ± 0.22	0.17 ± 0.28	Wilcoxon SRT	n.s.
	V_21_(%)	2.64 ± 2.17	3.95 ± 2.6	Wilcoxon SRT	<0.001
**Pelvic marrow**	D_mean_(cGy)	1235.7 ± 93.79	1325.9 ± 78.66	Wilcoxon SRT	<0.001
	V_10_(%)	85.53 ± 5.44	88.07 ± 5.24	Paired t-Test	<0.001
	V_15_(%)	66.54 ± 5.95	73.03 ± 4.96	Paired t-Test	<0.001
	V_25_(%)	34.31 ± 6.37	41.08 ± 6.16	Paired t-Test	<0.001
	V_5_(%)	1.30 ± 0.85	1.02 ± 0.82	Wilcoxon SRT	0.00218
**Femoral head_L**	D_mean_(cGy)	480.68 ± 77.79	552.53 ± 84.22	Paired t-Test	<0.001
	V_5_(%)	40.4 ± 9.48	44.17 ± 10.42	Paired t-Test	0.03001
	V_10_(%)	12.12 ± 4.13	16.78 ± 4.97	Paired t-Test	<0.001
	V_15_(%)	0.69 ± 0.84	3.17 ± 2.67	Wilcoxon SRT	<0.001
	V_12.5_(%)	3.92 ± 2.47	8.87 ± 4.04	Paired t-Test	<0.001
**Femoral head_R**	D_mean_(cGy)	466.2 ± 63.87	531.62 ± 70.08	Paired t-Test	<0.001
	V_5_(%)	36.34 ± 6.22	41.43 ± 7.96	Paired t-Test	0.005007
	V_10_(%)	12.15 ± 3.5	15.68 ± 4.44	Paired t-Test	<0.001
	V_15_(%)	0.87 ± 0.91	2.59 ± 1.9	Wilcoxon SRT	<0.001
	V_12.5_(%)	4.03 ± 2.34	7.52 ± 3.55	Paired t-Test	<0.001
	HI	0.12 ± 0.01	0.09 ± 0.02	Wilcoxon SRT	<0.001
	CI	0.91 ± 0.02	0.92 ± 0.01	Wilcoxon SRT	<0.001
	GI	2.96 ± 0.21	3.45 ± 0.19	Paired t-Test	<0.001
					

**Fig 2:**
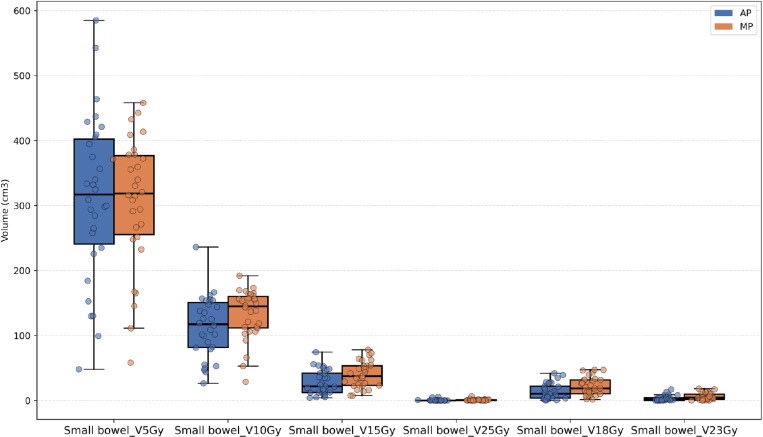
Jittered scatter box – plot of the absolute radiation volume received by the small bowel at different dose levels.

**Fig 3:**
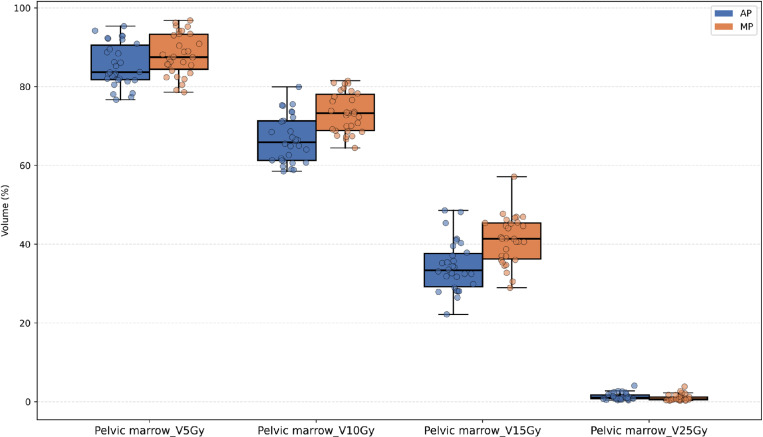
Jittered scatter box – plot of the percentage of radiation volume received by the pelvic marrow at different dose levels.

**Fig 4:**
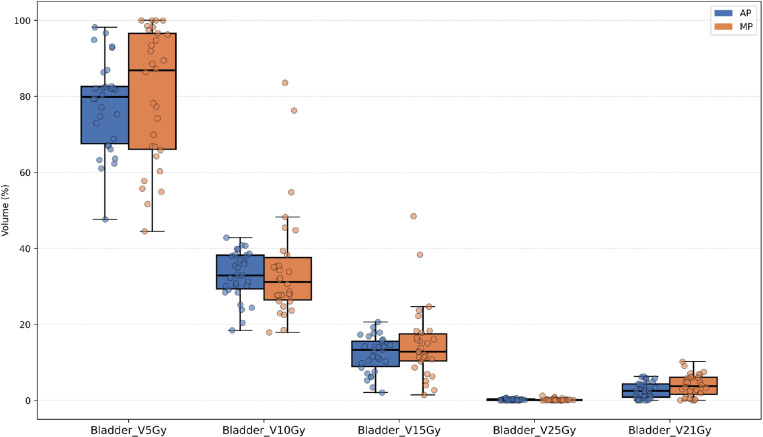
Jittered scatter box – plot of the percentage of radiation volume received by the bladder at different dose levels.

**Fig 5:**
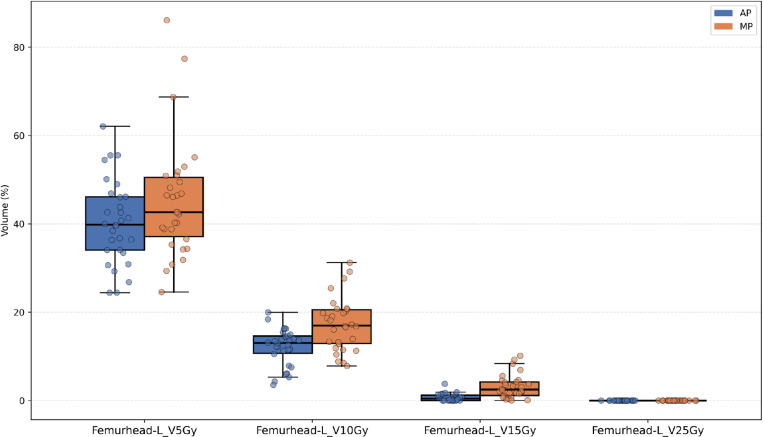
Jittered scatter box – plot of the percentage of radiation volume received by the Left femoral head at different dose levels.

**Fig 6:**
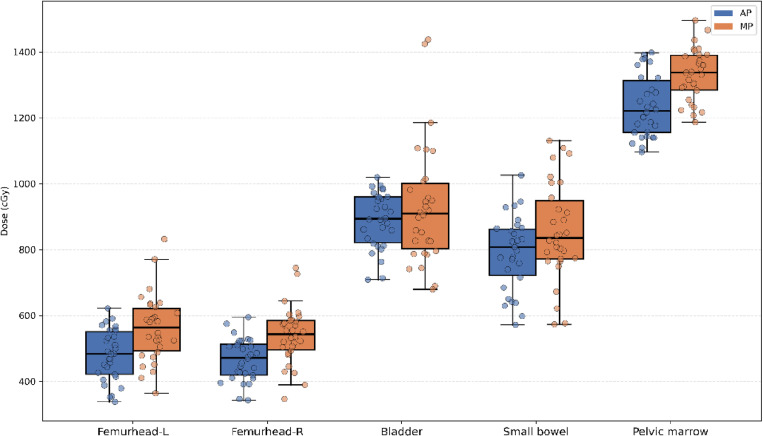
Box plot and jitter scatter plot of the mean dose to the OARs.

**Fig 7:**
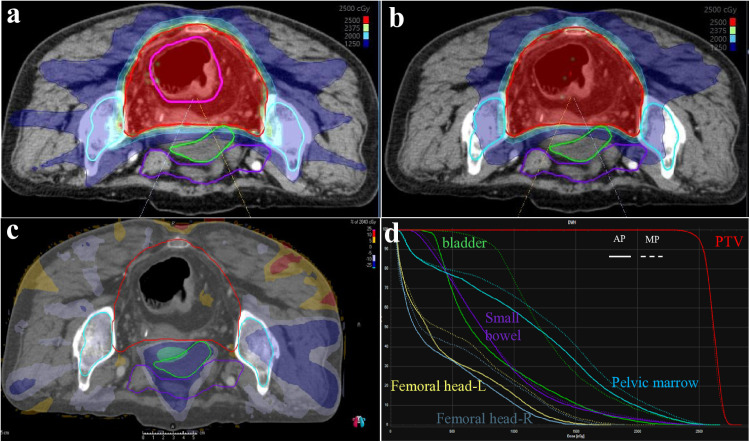
Dose distributions for MP. (a) and AP (b), dose differences (c) and DVH (d).

Except for the V5Gy dose level, the irradiated volume (cc) at most dose levels (V15Gy, V18Gy, etc.) for the small bowel in the AP was significantly lower than that in the MP, as shown in Fig 2. Figs 3–5 illustrate that, for the dose parameters (e.g., V10Gy, V15Gy) of the bladder, pelvic marrow, and femoral head, the dose volumes in the AP group were notably lower than those in the MP group, indicating that the AP more effectively spares these organs from irradiation. No significant difference was observed for the bladder V25Gy dose, which is considered clinically insignificant. The irradiated volume of the femoral head at V25Gy was 0. Fig 6 shows that the mean dose to five OARs in the AP group was significantly lower than in the MP group (e.g., small bowel 789.82 cGy vs. 855.68 cGy), indicating that the auto-planning can effectively reduce the dose to OARs while maintaining PTV coverage. For a selected patient, Fig 7 presents the dose distribution and dose-volume histograms (DVH) for both the APs and MPs, highlighting the differences in dose distribution between the two TPSs.

### Plan quality and QA

Regarding the MU, AP has approximately 4% fewer MU than MP on average. All plans achieved a gamma pass rate greater than 95% for the 3%−3 mm criterion in accordance with AAPM TG-119 recommendations. Gamma analysis was performed based on the composite delivery mode. No significant differences were observed in the validation results between the AP and MP, as shown in Table 2. The optimization time for AP, based on the RayStation script, ranges from 12 to 25 minutes, whereas manual planning using the Monaco TPS takes between 25 and 100 minutes, depending on planning complexity, computational server load, and patient-specific anatomical variations. Although this study is retrospective and does not allow for precise time analysis, an estimate by an experienced medical dosimetrist indicated that the RayStation AP time was (18.23 ± 3.59) minutes, while the MP time was (53.24 ± 21.11) minutes, resulting in a time savings of approximately 65%.

**Table 2:** Results of quality assessments for APs and MPs.

Parameters	AP	MP	*P*
**Complexity (mm **−**¹)**	0.105 ± 0.009	0.092 ± 0.009	<0.001
**MU**	2545.4 ± 270.21	2653.4 ± 179.37	0.0377
**Gamma pass rate (%)**	98 ± 1.7	98.3 ± 4.2	0.5

## Discussion

In this study, we developed a fully automated rectal cancer VMAT planning program on the Raystation TPS platform. This program simulates the manual planning process using a Python script and applies an iterative approach to evaluate and adjust the objective parameters during optimization, thereby automating the VMAT planning process [26,36,37]. Our results demonstrate that the AP method achieves plan quality comparable to or better than that of MP. As shown in Table 1 and Figs 2–4, the AP provides comparable target conformity, more rapid dose fall-off, and improved dose consistency, while also offering better protection for OAR and reducing their radiation dose.

The advantages of auto-planning quality for the pelvic region have been extensively documented in the literature. Kouta et al. [26] demonstrated that auto-planning significantly reduced the small intestine V30 (by 8.3%), V40 (by 4.3%), and the mean bladder dose (by 6.0 Gy), highlighting its advantages in sparing critical organs. Song et al. [22], utilizing the automatic VMAT plan optimizer (ARPO) developed with Pinnacle3 (Philips Healthcare, Fitchburg, WI, USA), found that, with similar PTV coverage, auto-planning provided superior protection for OARs, including the small intestine, bladder, and femoral head, particularly with a 2 Gy reduction in the mean dose to the small intestine. Liu et al. [21] developed and validated a fully automatic iterative planning (AIP) system based on the Varian Eclipse TPS (Varian Medical Systems, Palo Alto, CA), which showed that auto-planning reduced the mean dose to the femoral head, marrow, small bowel avoidance region, and bladder by 6.1%, 5.9%, 5.9%, and 1.8%, respectively, demonstrating significant advantages over traditional manual planning. Our results are consistent with these reports, and overall, auto-planning is either superior to or at least comparable with manual planning in terms of plan quality.

For patients with LARC undergoing preoperative SCRT, clinicians are particularly focused on the OARs. As indicated by the Stockholm III study, radiation-induced toxicity related to SCRT results in 7% of patients requiring preoperative hospitalization [38]. Furthermore, acute radiation-induced toxicity may adversely affect patients’ adherence to chemotherapy.

In AP V15, V18, V23, V25, and Dmax of the small bowel were significantly lower than those in MP, and Dmean decreased by 65 cGy. Considering that gastrointestinal toxicity is the most prevalent adverse effect during neoadjuvant treatment [39], which is a combination of radiation-induced and chemotherapy-related toxicities. Limiting radiation exposure to the bowel seems reasonable, as the volume of bowel tissue exposed to radiation can predict toxicity, even at low doses.

With respect to the pelvic marrow, V5, V10, V15, V25, and Dmean in AP were significantly lower than those in MP. Studies have indicated that lowering the bone marrow Dmean decreases the risk of hematologic toxicity [40]. Therefore, the AP achieves a 90 cGy dose reduction, and this resulting reduction in hematologic toxicity risk may positively influence the tolerance to subsequent chemotherapy.

The bladder’s V5, V10, V15, V21, and Dmean are lower than those achieved with MP. Previous studies have demonstrated that preoperative radiotherapy for rectal cancer may impact multiple aspects of urinary and sexual function [41]. Our study suggests that auto-planning, by providing better bladder sparing, may help mitigate adverse effects on both the urinary and reproductive systems.

Maintaining similar PTV coverage, AP increased the PTV Dmean by an average of 33 cGy (p < 0.001) and the maximum dose (D2%) by an average of 55 cGy. Thus, with similar PTV D98% and the same PTV D2%, AP is able to deliver a higher dose to the target. The HI (0.12) for AP was slightly higher than that of MP (0.09), while the GI (2.96 ± 0.21) was significantly superior to that of MP (3.45 ± 0.19), indicating that auto-planning achieves a steeper dose gradient outside the PTV, thereby enhancing sparing of OARs. Prioritizing OARs in relation to the PTV generally does not compromise tumor control [42], which is consistent with the principle of ‘moderate target coverage and strict protection of OARs’ in SCRT in our department.

Auto-planning has been reported to lead to an increase in total MUs when using the same TPS [10,21]. Moreover, Marrazzo et al. [10] found that, compared to manual planning with the Monaco TPS, the Pinnacle^3^ auto-planning module reduced the average number of monitor units (MUs) by 29%. In contrast, Han et al. [20] reported that auto-planning using the RayStation reduced total MUs by 20% compared to the Pinnacle^3^, demonstrating the advantages of RayStation in MU control.

In our study, auto-planning reduced MU by 4% compared to manual planning, and the complexity for AP (0.105) is higher than for MP (0.092). The RayStation TPS had 314 control points (one control point per 2 degrees), while the Monaco TPS uses variable control points (199.6 ± 21.39). The Monaco (one control point per variable degree) is automatically allocated by the TPS according to optimization requirements. Despite the higher complexity of AP, treatment delivery time was reduced.

The efficiency of auto-planning is a key feature [37,43–45], which aligns with the results of our study. This suggests that auto-planning not only significantly saves dosimetrists’ time but also provides superior solutions in complex planning scenarios.

Furthermore, the quality of results produced by AP is more consistent, reducing the influence of human factors on plan quality, thereby standardizing and streamlining the entire planning process. After automated optimization, if specific clinical requirements are needed, or if the dosimetrist is dissatisfied with the results, the generated plan can serve as a basis for further manual adjustments.

This study has two main limitations. First, although all MPs were generated by experienced dosimetrists, differences in the TPSs—particularly in optimization algorithms—as well as variations in dosimetrist expertise and institutional preferences, may have affected plan quality. Second, while the automated VMAT approach performed well in SCRT for LARC, its applicability to other anatomical sites remains unproven. Variations in anatomy, OAR constraints, and dose objectives could pose additional challenges. Importantly, this study aimed to evaluate the feasibility of replacing manual planning with auto-planning rather than directly comparing the two TPSs. Further research is needed to validate this method across diverse disease sites and to optimize algorithm parameters for broader clinical adoption.

## Conclusions

We developed a fully automated and feasible SCRT VMAT planning program for LARC. This program significantly enhanced plan quality and efficiency, while substantially reducing the dose to OARs.

## Supporting information

S1 File Auto-planning program. (ZIP)

S2 File Initial template. (TIF)

S3 File AP_dose_data. (ZIP)
