# Supplementary figures and images for: Automated VMAT planning for short-course radiotherapy in locally advanced rectal cancer
Source: PLoS One. 2025 Jun 9;20(6):e0325567. doi: 10.1371/journal.pone.0325567 (PMC12148120; doi:10.1371/journal.pone.0325567)

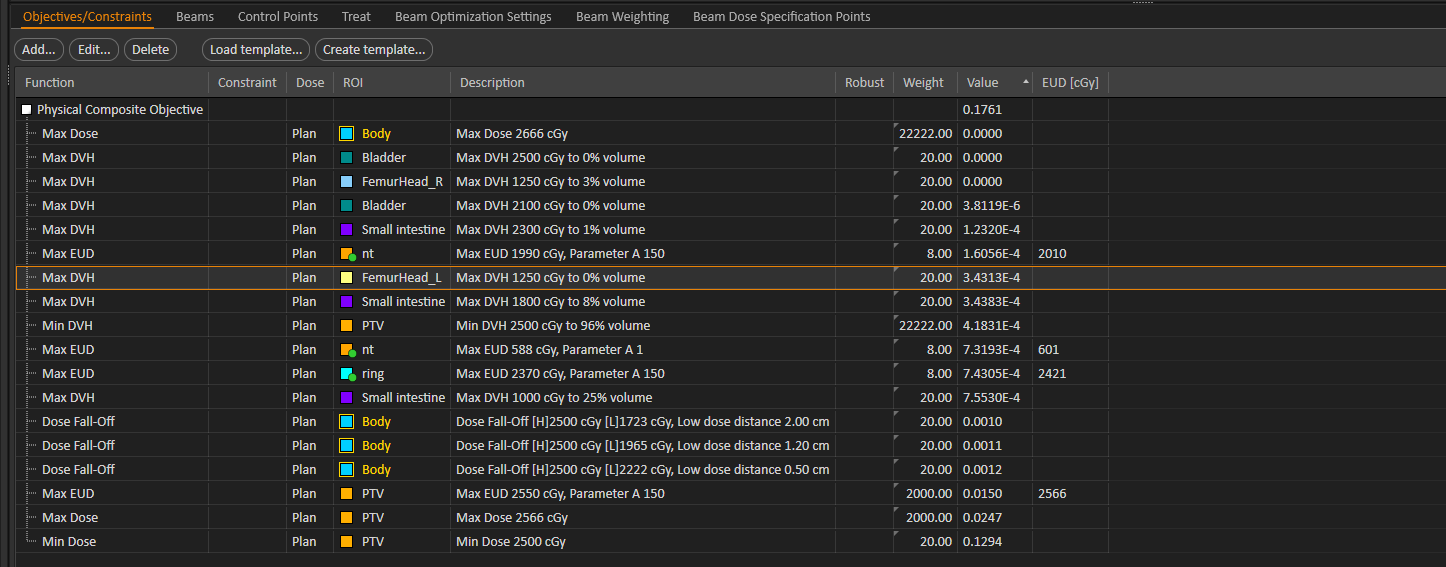

Supplement: S2 File — (TIF) [file pone.0325567.s002.tif]
